# Development and characterization of a wild emmer wheat backcross introgression population for hard winter wheat improvement

**DOI:** 10.1002/tpg2.70104

**Published:** 2025-09-01

**Authors:** John H. Price, Mary J. Guttieri, Moses Nyine, Eduard Akhunov

**Affiliations:** ^1^ Oak Ridge Institute for Science and Education (ORISE) Manhattan Kansas USA; ^2^ USDA‐ARS Hard Winter Wheat Genetics Research Unit, Center for Grain and Animal Health Research Manhattan Kansas USA; ^3^ Department of Plant Pathology Kansas State University Manhattan Kansas USA; ^4^ Wheat Genetics Resource Center Kansas State University Manhattan Kansas USA

## Abstract

Wild emmer wheat (*Triticum turgidum* subsp. *dicoccoides*) is the tetraploid progenitor of hexaploid bread wheat (*Triticum aestivum* L.) and is known to be a valuable source of genetic variation for wheat improvement. However, direct evaluation of wild emmer diversity for agronomic potential has limited value unless performed in the backgrounds of adapted cultivars. Here, we present a genetic characterization of a population of 1601 backcross recombinant inbred lines, with an average genome composition of 75% bread wheat and 25% wild emmer. Low‐coverage whole‐genome sequencing allowed introgressions and aneuploidies to be identified at a relatively low cost per sample. We identified a relatively large proportion of small introgressions (median length 38 Mb), and we found introgressions to be distributed across all chromosomes. Approximately 44% of genotyped progeny carried at least one aneuploidy, with monosomies being by far the most common. This population, which we have denoted as the Great Plains Wild Emmer/Hard Winter Wheat introgression population (GPWEW‐IP), is, to our knowledge, the first introgression population developed through the direct hybridization of wild emmer wheat and US‐adapted hard winter wheat. We believe that this population represents a valuable resource for wheat breeders and will accelerate the discovery and integration of useful variation from wild emmer wheat.

## Core Ideas

- Wild emmer wheat (WEW) is a valuable source of genetic variation for bread wheat improvement.
- To facilitate wheat breeding, we generated 1601 backcross lines of WEW introgressed into bread wheat backgrounds.
- WEW introgressions were well distributed across the genome, with negative selection at the Q domestication locus.
- Approximately 44% of individuals carried at least one aneuploidy as a result of this tetraploid × hexaploid cross.

## Plain Language Summary

The weedy wild ancestors of modern bread wheat are a reservoir of variation for resistance to diseases, tolerance to heat and drought stress, and nutritional quality. This natural variation was lost in the evolutionary cascade that led to modern bread wheat. This research integrates the variation found in wild emmer wheat, the ancestral source of two‐thirds of the genome of modern bread wheat. A set of 27 diverse wild emmer parents was mated to a set of five hard winter wheats. The hybrids of these matings were then pollinated with the original hard winter wheat parents. These matings were advanced by inbreeding to develop a library of 1601 lines that are 25% wild emmer by pedigree. These progeny were characterized by DNA sequencing. In these lines, wild emmer DNA generally was uniformly distributed across the genome. This set of breeder‐ready wheat materials, and the associated DNA sequence information, is a valuable new genetic resource for wheat improvement.

**Abbreviations**
RIL: recombinant inbred line
SNP: single‐nucleotide polymorphism
WEW: wild emmer wheat

## 1 INTRODUCTION

Bread wheat (*Triticum aestivum* L.) is an allohexaploid species, derived from the hybridization of domesticated allotetraploid emmer wheat (*Triticum turgidum* L. subsp. *dicoccon* (Schrank) Thell.) and diploid *Aegilops tauschii* (Coss) (Zohary et al., 2012). Emmer wheat itself derives from the domestication of wild emmer wheat (WEW) (*Triticum turgidum* L. subsp. *dicoccoides* (Körn. ex Asch. & Graebn.) Thell.) approximately 10,000 years ago (Zohary et al., 2012), likely in what is now Türkiye (Özkan et al., 2002). Wild populations of emmer may be found throughout the Levant, southern Türkiye, and east to western Iran (Özkan et al., 2011).

Wild emmer accessions have been considered a useful germplasm pool for wheat improvement since at least 1969, when a WEW accession known as G‐25 was identified as a source of stripe rust (*Puccinia striiformis* f. sp. *tritici*) resistance (Gerechter‐Amitai & Stubbs, 1970). This resistance was later identified as the seedling resistance gene *Yr15* (Klymiuk et al., 2018); to date, isolates of stripe rust virulent to *Yr15* have yet to be identified. Other disease resistance genes, including *Yr36* for stripe rust (Uauy et al., 2005) and *Pm41* for powdery mildew (Li et al., 2020), have subsequently been introduced into bread wheat from WEW, as well as the crucial stem rust resistance genes *Sr2* and *Sr13* from domesticated emmer wheat (Fraser, 2024).

In addition to disease resistance, WEW has been identified as a source of variation for nutritional quality (Chatzav, 2010), drought resistance (Peleg et al., 2007), and other traits of interest to wheat breeders. However, integration of useful variation not controlled by single, large‐effect loci into elite germplasm is slow, given the large number of alleles carried by any given wild emmer accession that are maladaptive in an agronomic setting. In addition, the relatively weedy habit of WEW and lack of key domestication traits, particularly non‐shattering rachis and free‐threshing glumes, limit the ability to truly evaluate the performance of WEW in an agronomic setting and to identify variation that could improve bread wheat performance.

One solution to allow the discovery of favorable alleles derived from unadapted germplasm is the development of backcross introgression lines (Ali et al., 2006) with elite recurrent parents that are well adapted to a target environment. By identifying inbred introgression lines, which display an improvement for a trait of interest relative to their recurrent parent, useful sources of variation derived from the unadapted source may be uncovered. If the recurrent parent is an elite cultivar or breeding line, the inbred introgression line immediately then serves as a useful crossing parent for breeders. In addition, such populations function as biparental mapping populations, allowing for the mapping of quantitative trait loci, which confer useful genetic variation.

We have applied this approach to WEW, developing a large backcross introgression population, known as the “Great Plains Wild Emmer/Hard Winter Wheat introgression population (GPWEW‐IP)”, by crossing 27 wild emmer accessions into a set of five elite hard winter wheat lines well adapted to the US Central Great Plains. Here, we use a whole‐genome skim sequencing method to present a genetic characterization of introgression patterns and aneuploidy in this population, laying a foundation for efforts to identify and deploy useful allelic variation from this population in wheat improvement programs.

## 2 MATERIALS AND METHODS

### 2.1 Germplasm development

#### 2.1.1 WEW parent material

**FIGURE 1:**
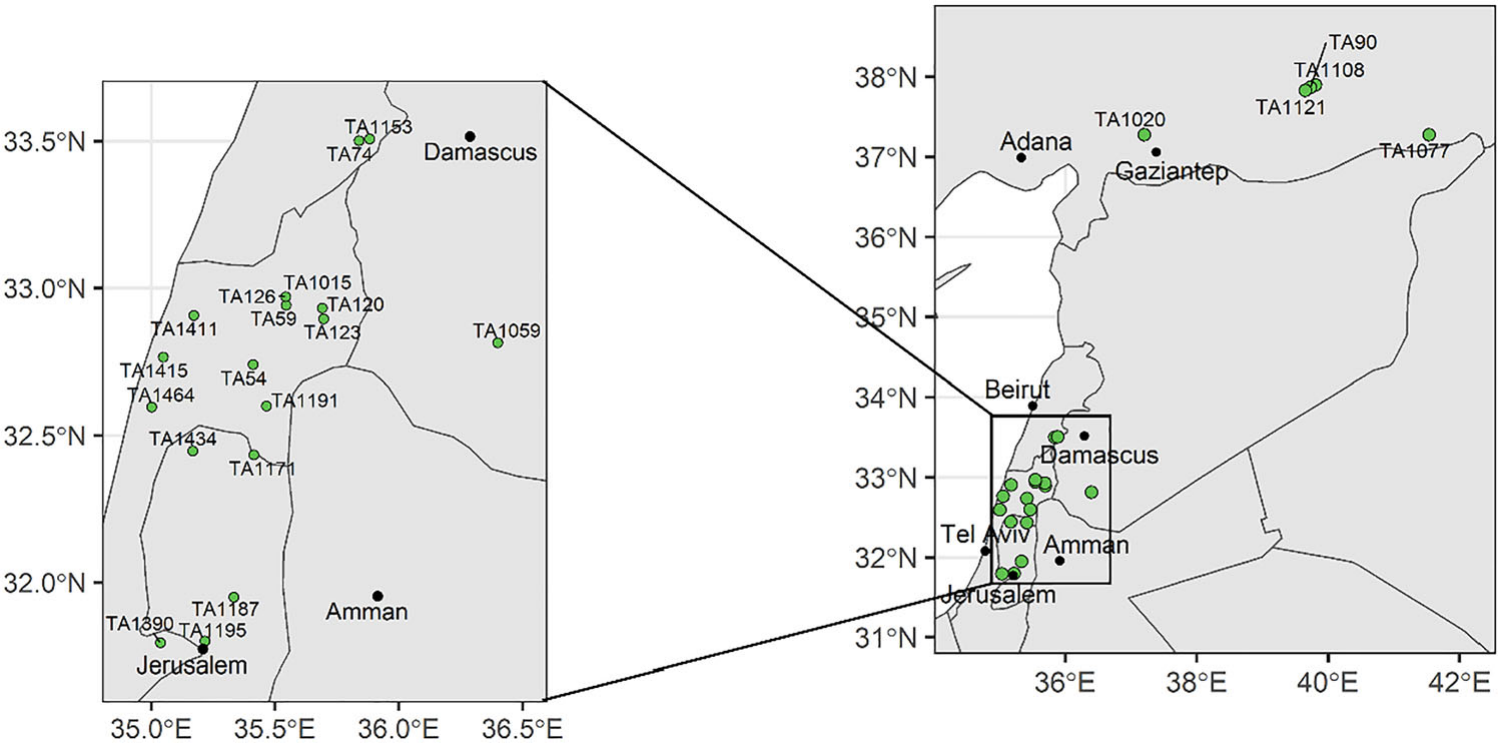
Origin of wild emmer wheat (WEW) parents used to develop this population.

Twenty‐seven wild emmer accessions were selected from the germplasm collection housed in the Kansas State University Wheat Genetic Resources Center (WGRC). These accessions, collected across the native range of WEW (Figure 1), were previously identified as a representative sample of the WGRC WEW collection (Yadav et al., 2023). Full passport information may be found in Table S1.

#### 2.1.2 Selection of recurrent parents

Five hard winter wheat lines with adaptation to the US Great Plains region were selected to serve as recurrent parents for this population. KanMark (PI 675456) is a hard red winter wheat variety developed by Kansas State University. KS090387K‐20 is a hard red winter wheat breeding line developed by Kansas State University having the pedigree Winterhawk (PI 652927)/KS011020‐6//Hitch (PI 655954). Monarch (PI 691606) is a hard white winter wheat released by Colorado State University, Byrd (PI 664257) is a hard red wheat released by Colorado State University (Haley et al., 2012), and Freeman (PI 667038) is a hard red wheat released by the University of Nebraska (Baenziger et al., 2014). KanMark and KS090387K‐20 were selected as recurrent parents on the basis of their resistance to lodging and their desirable end‐use quality. KanMark carries the 1BL.1BS‐3Ae#1 translocation that confers *Lr24/Sr24*, and KS090387K‐20 carries the 2N^v^S translocation conferring *Yr17*/*Lr37*/*Sr38*. These translocations are expected to restrict recombination with WEW on 1BS and 2AS, respectively, and thus KanMark and KS090387K‐20 are complementary. KanMark and KS090387K‐20 are well adapted from central Kansas south through Oklahoma and into Texas. Monarch was selected for its resistance to lodging, its susceptibility to field populations of leaf rust, and its white seed color, which may broaden the utility of introgression germplasm. Byrd and Freeman were selected based on their susceptibility to field populations of stripe rust. Additionally, they are photoperiod sensitive and later maturing, extending the region of adaptation into Nebraska and Colorado.

#### 2.1.3 Crossing and population advancement

Initial crosses were made in the greenhouses in the fall of 2017 and spring of 2018, with manually emasculated hexaploid wheat parents used as females and WEW parents used as males. All F_1_ seeds were planted from each cross, and F_1_ progeny, which survived to flowering, were backcrossed in the greenhouse to the hexaploid wheat parent, with the manually emasculated F_1_ plant used as the female. The BC_1_F_1_ seeds were planted and grown to maturity in the greenhouse. When possible, five BC_1_F_2_ seed from 10 unique BC_1_F_1_ plants were planted into 50‐well flats, and plants were grown to maturity on capillary mats in the greenhouse. Where fewer than 10 BC_1_F_1_ plants produced seed, the fifty wells were distributed approximately equally among the available BC_1_F_1_‐derived families. From these plants, progeny were advanced in the greenhouse by single‐seed descent through the F_5_ generation. Figure S1 outlines this breeding scheme. Seed (BC_1_F_5:6_) from each plant was then multiplied in Yuma, AZ, to provide seed (BC_1_ F_5:7_) for subsequent evaluation.

**Core Ideas**

- Wild emmer wheat (WEW) is a valuable source of genetic variation for bread wheat improvement.
- To facilitate wheat breeding, we generated 1601 backcross lines of WEW introgressed into bread wheat backgrounds.
- WEW introgressions were well distributed across the genome, with negative selection at the Q domestication locus.
- Approximately 44% of individuals carried at least one aneuploidy as a result of this tetraploid × hexaploid cross.

### 2.2 Genotyping

Leaf tissue was collected from individual greenhouse‐grown BC_1_F_5:6_ plants and genotyped using a whole‐genome skim sequencing approach, as in Adhikari et al. (2022). Briefly, genomic DNA was extracted from lyophilized leaf tissue using a BioSprint DNA kit (Qiagen Inc.). Low‐volume, highly multiplexed libraries were then created, using llumina Tagment DNA TDE1 Enzyme and Buffer Kits (Illumina Tagment DNA TDE1 Enzyme and Buffer Kits, Illumina, Inc.). Libraries were PCR amplified, dual‐indexed, normalized to 15 µL at 6 ng/µL, and pooled. Paired‐end sequencing was done by Psomagen with Illumina NovaSeq 6000 or HiSeq X Ten.

Separately, deeper whole genome sequence data were collected for each recurrent and wild emmer parent, using the TruSeq DNA PCR‐Free protocol for library preparation (Illumina, Inc.). All sequence data are deposited in the NCBI SRA (PRJNA1260574). For the bread wheat parent KanMark, publicly available whole‐genome sequence data published to the NCBI SRA SRX11411614 was used, as opposed to resequencing this line. All subsequent analysis steps were identical for KanMark and all other parents.

### 2.3 Analysis

#### 2.3.1 Variant calling

Variants were detected in whole‐genome skim sequence data, following a pipeline adapted from Adhikari et al. (2022). Adaptor sequences were first trimmed from demultiplexed reads, using the program “fastp” (Chen et al., 2018). These reads were mapped to the Chinese Spring version 2.1 bread wheat reference genome using HISAT2 (Kim et al., 2019; Zhu et al., 2021). The resulting sam files were then filtered to remove all reads that were not concordantly mapped to a unique position (Adhikari et al., 2022). Then, single‐nucleotide polymorphism (SNP) variants were jointly called from all recurrent and wild emmer parents, using BCFtools “mpileup” and “call” (Danecek et al., 2021). The resulting VCF file was converted to a tab‐delimited SNP positions file to create a target file for progeny genotyping (Adhikari et al., 2022). Because calling genotypes for all 1601 recombinant inbred line (RIL) lines at each of these sites produced formidably large genotyping files, hindering down‐stream analysis, a reference subset was developed of only those SNP loci that were successfully called and homozygous in all 32 parents—this also helped ensure SNP quality. Variants were then called at these positions in the introgression lines, again using BCFtools “mpileup” and “call.”

#### 2.3.2 Identifying aneuploidy

To identify aneuploidy and chromosomal deletion events, read depth was calculated at each 1 Mb bin as in Adhikari et al. (2022). Briefly, for each sample, the UNIX tools “grep” and “awk” were used to count the number of unique, concordantly aligned reads in each bin, which was then divided by the average number of reads per bin for that sample. For each sample, the mean and standard deviation of normalized read depth was then calculated for each arm of each chromosome. Chromosomes where both arms had an average normalized read depth below 0.2 were considered nullisomic, both arms between 0.2 and 0.7 were considered monosomic, and above 1.25 and below 1.75 were considered trisomic.

#### 2.3.3 Measuring diversity among parents

For each pair of parental lines, pairwise nucleotide diversity was calculated for each 1 Mb bin as number of differing homozygous SNPs/total number called SNPs. The mean pairwise nucleotide diversity was then calculated at each 1 Mb bin across all pairs of bread wheat lines, across all pairs of WEW lines, and across all bread wheat/WEW pairs.

#### 2.3.4 Identifying introgression segments

Introgression regions were identified in progeny through comparison with parental lines, using a custom R script. For each wild emmer x hexaploid wheat parental combination, all loci which were heterozygous in either parent or where the allele call did not differ between the two parents were removed. Then, for each progeny line in the family, the number of alleles derived from each parent was counted in 1 Mb bins. The ratio of wild emmer to hexaploid wheat unique alleles was used to identify the bin as homozygous for one of the parental haplotypes or as heterozygous.

In practice, the low density of sequence data left many 1 Mb bins either completely unassigned or ambiguous. To impute values for these bins, a moving average wild emmer allele proportion was calculated for each bin, based on the total count of wild emmer and hexaploid wheat‐specific alleles in the 5 Mb before and after each bin. This approach has the effect of creating ambiguity as to the exact location of the recombination event but was considered the optimal imputation approach. The population mean of this value for each bin across the genome was then reported as mean wild emmer allele proportion.

#### 2.3.5 Identifying introgression segments

To develop summaries of introgression segment length, introgression segments were identified as consecutive runs of bins where the 10 Mb moving average wild emmer allele proportion did not fall below 0.9 or the 40 Mb moving average wild emmer allele proportion did not fall below 0.63. The selected threshold values excluded heterozygous sites from this summary and excluded all sites with a normalized read depth below 0.5 or above 1.5 in order to more confidently identify introgression segments and therefore better estimate length. Introgression segments shorter than 1 Mb were excluded as being biologically implausible. The probability of introgression in each bin of the wheat genome for the RILs in this study will be available at Ag Data Commons (DOI: 10.15482/USDA.ADC/28629710).

#### 2.3.6 Calculation of recombination rate

To quantify recombination rate, the Pearson correlation coefficient was calculated between the wild emmer allele proportion of each pair of 1 Mb bins across each chromosome. The recombination landscape across each chromosome was then visualized by counting, for each bin, the number of other bins with which it had a recombination frequency at or below 0.1 (Pearson's correlation coefficient ≥0.9). The maximum value of this metric for each chromosome was used as a measure of the length of the centromeric region of repressed recombination for that chromosome.

## 3 RESULTS

### 3.1 Genotyping

In total, 1601 BC_1_F_5:6_ individuals, as representatives of 1601 BC_1_F_5_‐derived recombinant inbred lines, were genotyped using whole‐genome skim sequencing. On average, 966,035 paired‐end 150 bp reads were obtained per individual, resulting in an average genome coverage of 0.0099×. Of these, 69.5% of the read pairs mapped concordantly and uniquely to the reference genome.

In addition, whole‐genome sequencing was conducted for four bread wheat parents (excluding KanMark, which was sequenced as part of a previous experiment) and 27 WEW parents. An average of 587,074,432 paired‐end 150 bp reads were obtained per individual for hexaploid wheat parents, and 358,732,400 for WEW parents, for an average genome coverage of 6× for hexaploid bread wheat parents, and 5.35× for tetraploid WEW parents. Of these, 76.9% and 69.9% of read pairs mapped concordantly and uniquely to the Chinese Spring v2.1 bread wheat reference genome for hexaploid wheat parents and tetraploid WEW parents, respectively.

In the parental population, a total of 368,839,045 SNP sites were called across the A and B subgenomes. Calling genotypes for all 1601 progeny individuals at all segregating sites produced formidably large genotyping files, hindering down‐stream analysis. To overcome this challenge, a reference subset was developed consisting of only those SNP loci that were successfully called and homozygous in all 32 parents—this also helped ensure SNP quality. This reference subset consisted of 211,095,653 SNP sites across the A and B subgenomes, with an average of 480,142 of these SNP markers successfully called per progeny individual. Of these, an average of 125,593 SNP loci were homozygous in both parents of the line and differed between the two parents of the line. These SNPs were considered informative.

### 3.2 Diversity among parental lines

**FIGURE 2:**
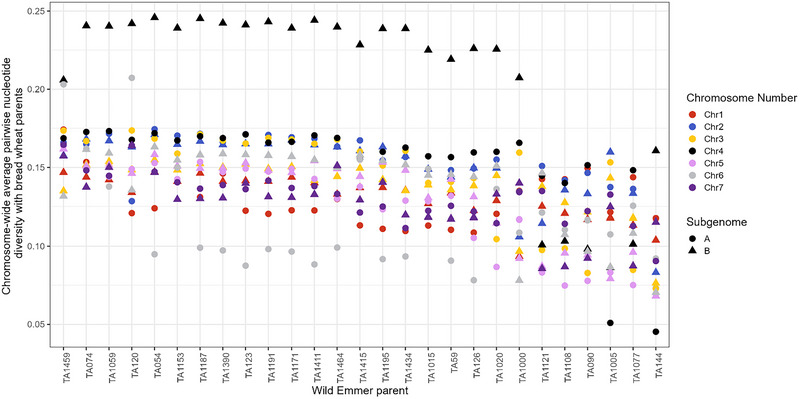
Mean pairwise whole‐chromosome nucleotide diversity between each wild emmer wheat (WEW) line and the five bread wheat lines. Pairwise nucleotide diversity was calculated for each 1 Mb bin as number of differing homozygous single‐nucleotide polymorphisms (SNPs)/total number called SNPs.

Across the majority of wild emmer parents, chromosome 4B showed the highest level of divergence from the domesticated wheat parents, with an average of 23.2% of homozygous SNPs differing between the WEW and bread wheat lines in pairings where it was the chromosome with the highest average distance (Figure 2). The exception to this pattern was a set of four WEW accessions collected in eastern Türkiye: three of these lines showed the greatest differentiation from bread wheat on chromosome 2A, and one on 3A. Some differentiation between WEW and bread wheat on chromosome 2A is likely driven by the presence of the 2N^v^S translocation, carried by the recurrent parents Monarch, KS090387K‐20, and Freeman. In the majority of parental pairings, chromosome 6A showed the lowest level of divergence between WEW and bread wheat, with an average of 9.7% of homozygous SNPs diverging between the WEW accessions and bread wheat lines in the subset of pairings where it was the chromosome with the lowest pairwise distance.

**FIGURE 3:**
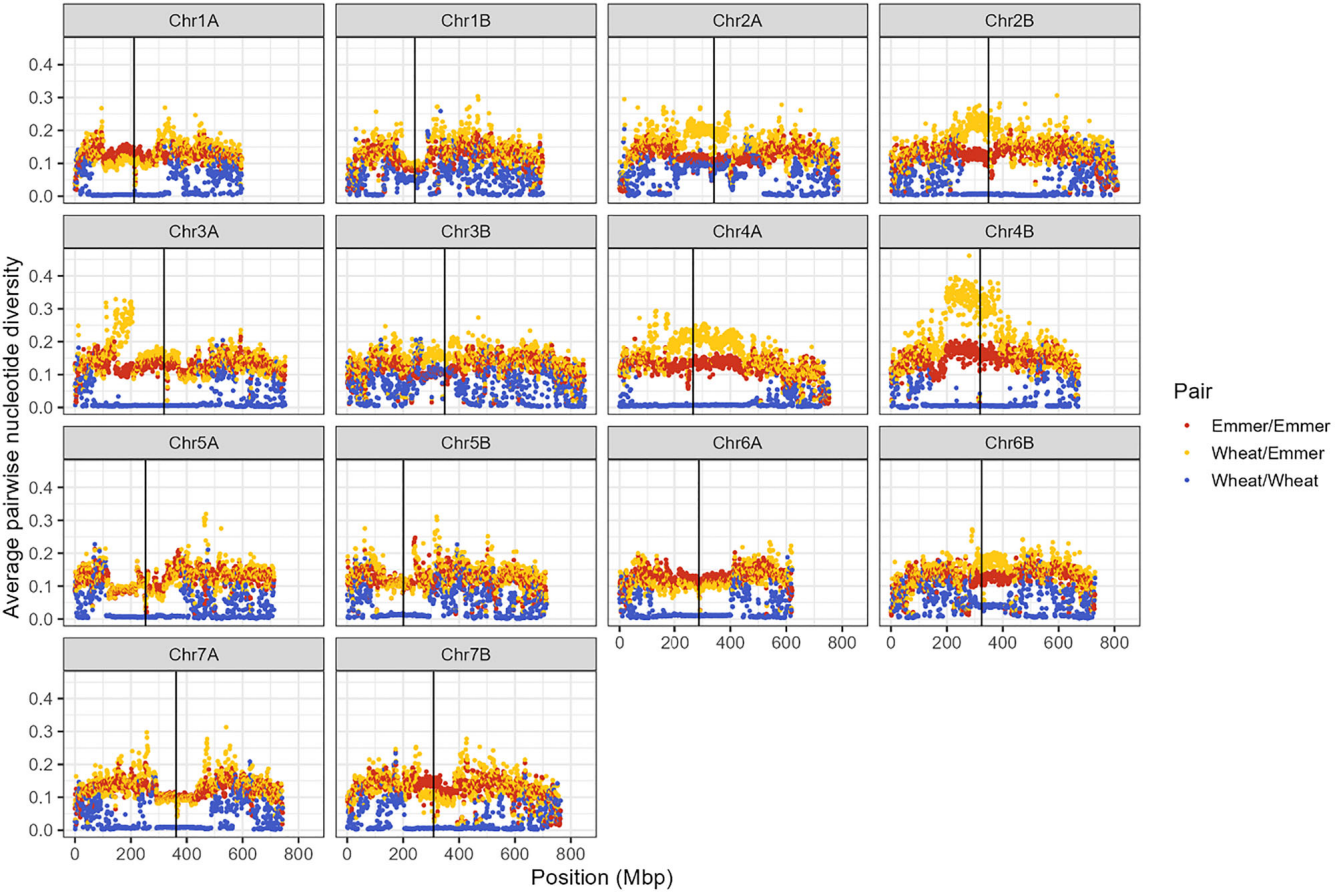
Average pairwise nucleotide diversity for each 1 Mb bin, calculated as Number of segregating single‐nucleotide polymorphism (SNP) sites between two individuals/total SNP sites in the population, averaged for all emmer/emmer, wheat/wheat, and wheat/emmer pairs of parental lines. Vertical lines denote centromeres, from Walkowiak et al. (2020).

Within chromosomes, the expected pattern of reduced diversity in centromeric regions was observed (Figure 3). This was especially pronounced among wheat/wheat comparisons, with no diversity observed among the five bread wheat recurrent parents in the centromeric regions of 10 out of 14 tetraploid subgenome chromosomes (Figure 3).

### 3.3 Patterns of introgression in progeny lines

**FIGURE 4:**
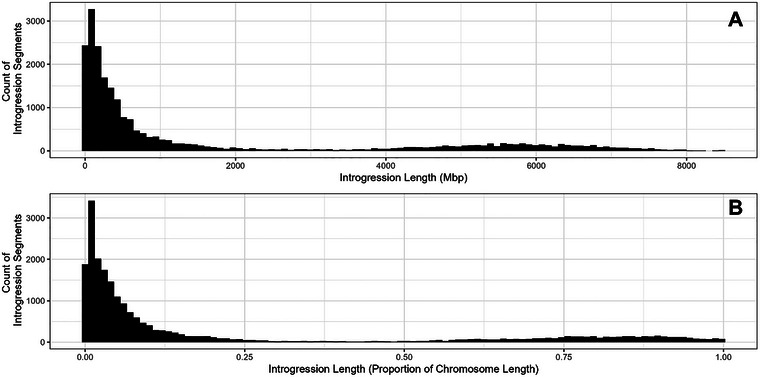
Distribution of introgression length segments across the population, by physical length (A) and length as a proportion of chromosome length (B).

On average, eleven of the fourteen A and B genome chromosomes per BC_1_F_6_ individual were observed to carry at least one introgression from emmer (here defined as at least one bin with a 10 Mb moving average wild emmer allele proportion above 0.7, irrespective of average read depth). The minimum number of chromosomes with an introgression was three, and 6% of individuals carried introgressions on all 14 A and B chromosomes. Of the 14 tetraploid genome chromosomes, Chromosome 4B was the least likely to contain an introgression segment, with 67% of individuals carrying an emmer introgression, and Chromosome 7B was the most likely, at 85% of individuals (Tables S2 and S3). The mean and median introgression lengths were 157 Mb and 38 Mb, respectively. Generally, introgression length was skewed toward small segments (Figure 4).

**FIGURE 5:**
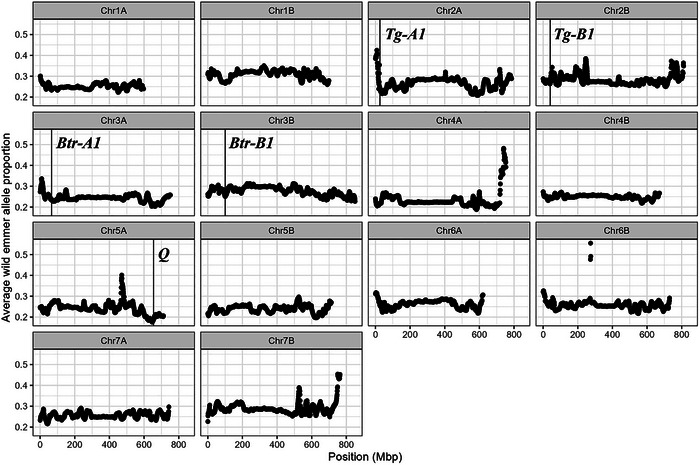
Average proportion of wild emmer alleles in each 1 Mb bin across the introgression population. Vertical lines indicate the denoted domestication gene. *Tg‐A1* and *Tg‐B1*: Tenacious glume A/B; *Btr‐A1* and *Btr‐B1*: Brittle rachis 1 A/B; *Q*: Q‐locus (free threshing, shattering, head shape). All positions for Chinese Spring v2.1 reference genome.

Across this population, the mean observed proportion of emmer alleles across the genome was 0.261, slightly higher than the expected proportion of 0.250. This proportion varied widely: across all 1 Mb bins, the highest observed 10 Mb sliding window emmer allele proportion was 0.555, and the lowest was 0.176 (Figure 5). The region with the lowest average proportion of emmer alleles, from 620 Mb to 656 Mb on Chromosome 5A, includes the key Q domestication locus (Faris et al., 2003). The wild allele at this locus results in a high level of glume adherence to the seed (Z. Zhang et al., 2011); extracting seeds from the head for planting may have resulted in embryo damage, causing inadvertent selection against wild‐type Q individuals during population advancement.

### 3.4 Aneuploidy

**FIGURE 6:**
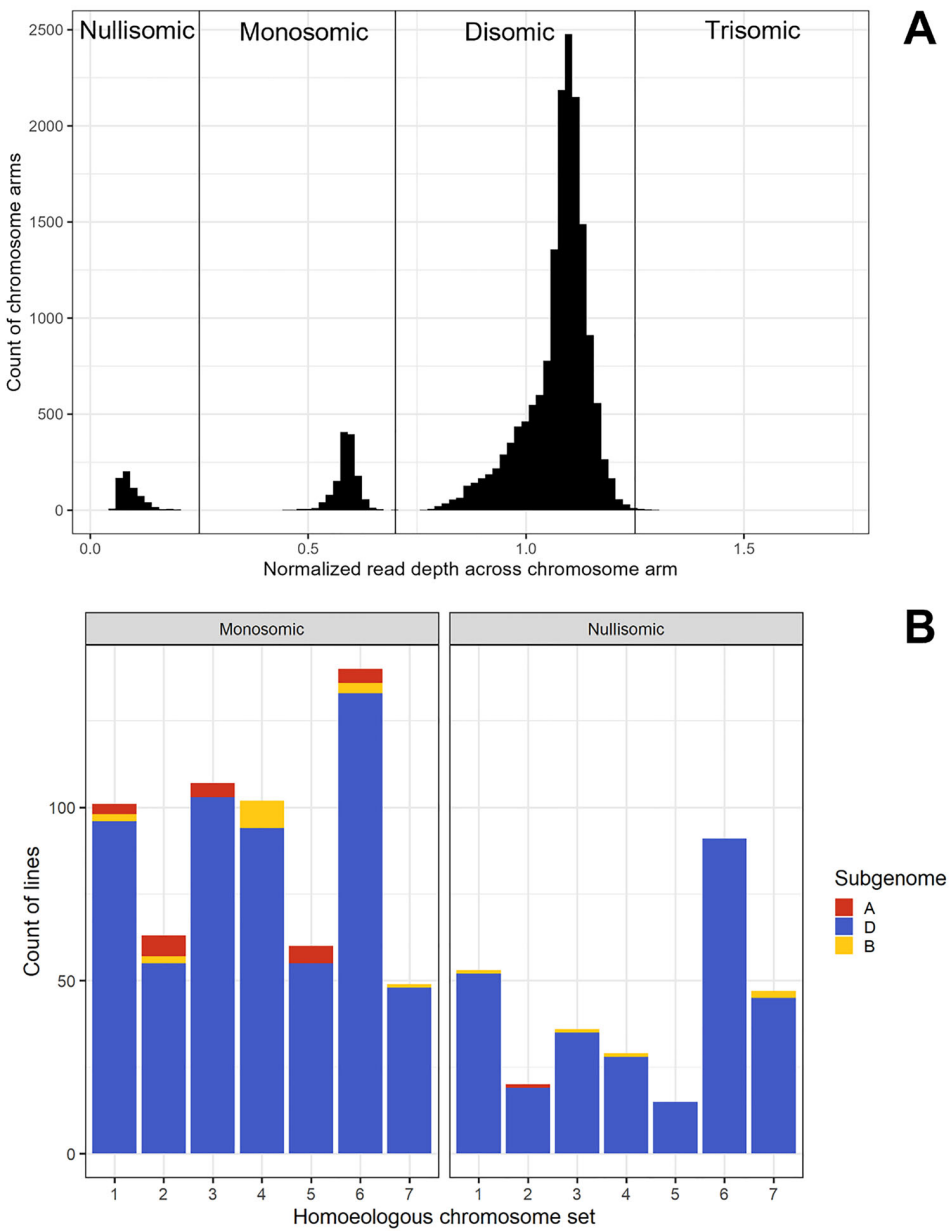
(A) Distribution of mean normalized read depth for chromosome arms without major deletion or duplications within the chromosome arm (defined here as a standard deviation for normalized read depth below 0.3). Vertical lines indicate threshold values for nullisomies, monosomies, disomies, and trisomies. Note that both chromosome arms needed to fall into the same category for the chromosome to be classified in that category, otherwise. the chromosome was not categorized—thus, the presence of a trisomic arm does not indicate a trisomic chromosome. (B) Count of full‐chromosome monosomies and nullisomies by chromosome. Population size = 1601 BC_1_F_6_ individuals.

Approximately 44% of BC_1_F_6_ individuals showed aneuploidy in at least one chromosome. At least one whole‐chromosome deletion was identified within 216 individuals (13.5% of the population) (Figure 6). Among these chromosome‐deletion individuals, 97.9% of the whole‐chromosome deletions occurred in the D subgenome (Figure 6). Nine individuals (0.56%) experienced a complete loss of the D subgenome. Chromosome 6D was by far the most likely to experience a complete nullisomy, accounting for 31.9% of D‐genome nullisomies (Figure 6). Chromosome 5D was the least likely, at just 5.3% of D‐genome nullisomies.

At least one whole‐chromosome monosomy was observed in 538 individuals (33.7%) (Figure 5). Again, a high percentage of these events, 93.9%, occurred in the D subgenome (Figure 6). Chromosome 6D was the most likely to be monosomic, with 22.9% of D subgenome monosomies occurring there, while 7D was the least likely, with 8.2% of D subgenome monosomies. Finally, only nine individuals (0.564%) showed at least one whole‐chromosome trisomy. Eight of these events occurred on Chromosome 4B, and one occurred on Chromosome 4D.

### 3.5 Patterns of recombination

**FIGURE 7:**
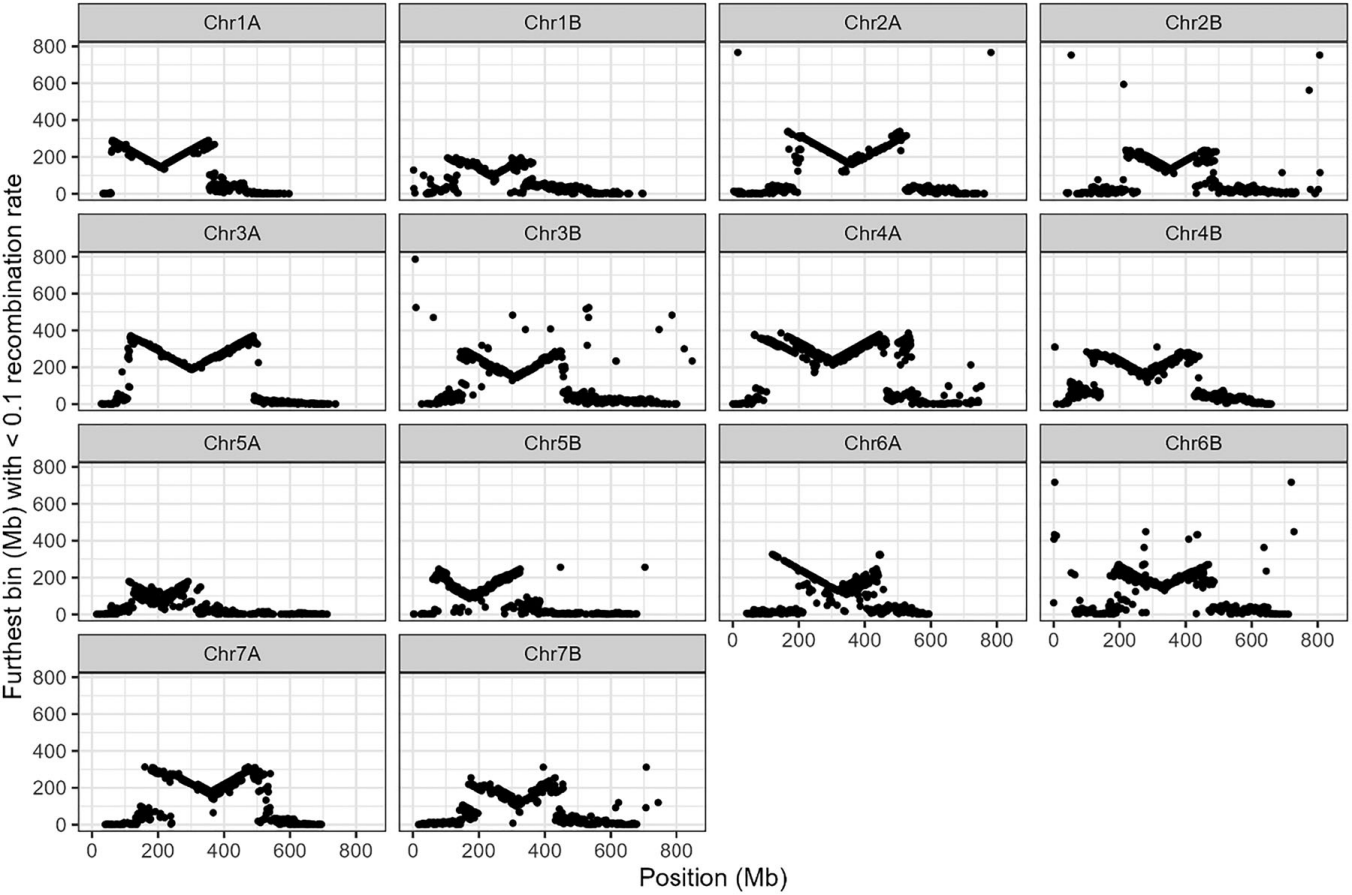
Length of recombination suppression across each chromosome. Each point represents the 1 Mb bin at the position indicated on the *x*‐axis. The *y*‐axis indicates, for that bin, the distance to the furthest bin with which the bin noted on the *x*‐axis has a recombination frequency at or below 0.1.

Recombination frequency, measured both as the proportion of individuals with a recombination event in a given bin and in the length of highly linked segments, varied across and between chromosomes. As expected, recombination was suppressed in the centromeric and pericentromeric regions, and much more frequent in the distal regions of chromosomes (Figure 7). However, the length of pericentromeric suppression varied widely by chromosome, from 25.1% of the chromosome length (179 Mb) for chromosome 5A to 52.5% of the chromosome length (326 Mb) for chromosome 6A. For six of the seven homologous chromosome sets, the A subgenome chromosome had a longer region of pericentromeric recombination suppression than its B subgenome counterpart, with 5A/5B being the lone exception.

## 4 DISCUSSION

### 4.1 Patterns of diversity among parental lines highlight genomic regions for exploration

A number of chromosomes showed virtually no diversity among the five wheat parents in the centromeric and pericentromeric regions. While these regions are unlikely to be gene‐rich (Zhao et al., 2023), they still merit further exploration. Notably, while the expected recombination suppression in the pericentromeric and centromeric regions is observed, there does not appear selection against individuals carrying WEW‐derived centromeric regions (Figure 3).

As an example, chromosome 4B displayed a particularly high degree of divergence (Figures 2 and 3). This is likely in part driven by a selection bottleneck in modern North American wheat germplasm with the introduction of the *Rht‐B1b* reduced height allele, located on 4B near the centromere (Ellis et al., 2002). The five hexaploid wheat lines used as recurrent parents for this population carry the reduced height allele for this locus. Further studies with non‐*Rht*‐*B1b* bread wheat lines could uncover different diversity patterns. Nonetheless, identifying RILs with close recombination events between the *Rht‐B1b* allele and wild emmer genomic segments would be an interesting future project.

The overall pattern of longer stretches of pericentromeric recombination suppression in the A subgenome relative to the B subgenome is interesting and has been observed in purely tetraploid wheat populations (Maccaferri et al., 2015), as well as hexaploid wheat populations (Jordan et al., 2018), indicating that the phenomenon is not merely an artifact of interploidy crossing. This population could serve as a useful tool for future explorations of A and B subgenome chromosome dynamics in wheat, particularly in disentangling patterns that are specific to domesticated wheat to those that are intrinsic to the ancestral A and B subgenome per se.

### 4.2 Numerous small introgression exist, which could facilitate integration into breeding populations

The median introgression size in this population was 38 Mb, with 23.1% of introgressions below 10 Mb, though further work will be necessary to differentiate true short introgression segments from bioinformatic artifacts. The presence of these small introgression segments is encouraging; for any given beneficial allele discovered, it is likely that several RILs may be identified, which carry this beneficial allele on a relatively small introgression segment. This in turn will reduce the number of crosses required to break any unfavorable linkages with targeted WEW genomic segments, speeding the process of moving useful alleles forward in the breeding pipeline.

### 4.3 Patterns of aneuploidy in wheat interploidy crossing

This population helps to highlight the incredible lability of the hexaploid wheat genome, with 44% of individuals carrying at least one whole chromosome nullisomy, monosomy, or trisomy as a result of the sequential hexaploid × tetraploid and pentaploid × hexaploid crosses, which were the foundations of this population.

In interpreting patterns of aneuploidy in this population, it is important to remember that these observations are derived from sequencing a single BC_1_F_6_ individual for each RIL. The presence of aneuploidy in these individuals does not mean that the aneuploidy is expected to be fixed in the RIL. An RIL showing monosomy in this population will likely contain disomic, monosomic, and possibly nullisomic and trisomic individuals (Riley & Kimber, 1961).

A relatively high proportion of monosomes, even after several generations of self‐pollination, has also been observed in wheat synthetic hexaploids generated by diploid × tetraploid crossing (H. Zhang et al., 2013). In that study, spontaneous monosomies occurred in the progeny of euploid individuals. It is likely that the same phenomena accounts for some proportion of the monosomes observed in the present study, rather than monosomies only occurring through maintenance from the original pentaploid. Indeed, this phenomenon is also known to occur even in stable inbred hexaploid wheat cultivars (Riley & Kimber, 1961).

The vast majority of nullisomies and monosomies were observed in the D subgenome (Figure 5). This supports previous observations from hexaploid × tetraploid crossed (Martin et al., 2011). However, our study confirms that a preference for the elimination of D subgenome chromosomes, relative to A or B subgenome chromosomes, is also present in pentaploid × hexaploid populations. This is in contrast to the relative stability of the D subgenome in the generation of synthetic hexaploids (H. Zhang et al., 2013).

The maintenance and stability of these aneuploidies in the RILs, as compared to the genotyped parent individual, is an open question, which will require further genotyping work to address. In increase nurseries, those lines that were subsequently identified as having a monosomic genotyped representive were noted to be segregating for a visually apparent phenotype (such as height, maturity, vigor, or head color) at a significantly higher rate than euploid lines (13.5% compared to 7.44%, *p* = 0.0002 for a chi‐square test), likely reflecting, in some cases, segregation for the aneuploid chromosome. In some cases, these plots were rogued to promote phenotypic uniformity, as it was assumed this segregation represented remnant heterozygosity. No traits could readily be associated with nullisomy in post hoc analysis of observations from the increase nursery; however, further work may uncover useful phenotypic variation associated with these aneuploidy events.

### 4.4 This population provides a wide range of emmer diversity in a hexaploid wheat background

At any given locus in this set of 1601 RILs, an average of 390 lines in the GPWEW‐IP carry an emmer introgression, representing all 27 WEW donor parents. In many cases, the same WEW allele may be replicated in several different bread wheat recurrent parent backgrounds, increasing available options for phenotypic screening.

This population enriches and complements the growing number of hexaploid, wild‐emmer derived populations available for wheat breeders. Previously, several synthetic hexaploid populations, which recreate the initial evolution of bread wheat by hybridizing tetraploid wheat (either WEW, durum, or domesticated emmer wheat) with diploid *A. tauschii*, have been introduced as valuable breeding resources (Gorafi et al., 2025; Rosyara et al., 2019). Our population complements these efforts by deriving a domesticated D subgenome from bread wheat, and developing A and B subgenomes that contain both bread wheat and WEW. These factors result in a population which is much closer to domesticated wheat than a synthetic population, meaning WEW allelic variation can be more rapidly incorporated into elite cultivars.

Direct crossing of WEW with bread wheat to develop introgression lines is far less common; most other populations have consisted of small number of WEW lines crossed with Israeli spring wheats and/or using a durum bridge (Chandrasekhar et al., 2017; Rong et al., 2000; Uauy et al., 2005). To our knowledge, the population we have developed is the first created through the direct hybridization of WEW and US‐adapted hard winter wheat and uses a wider range of WEW diversity than generally available. Therefore, we foresee the GPWEW‐IP serving as a valuable resource for wheat breeders across the Great Plains. Indeed, a number of projects are underway to uncover sources of disease or pest resistance, drought tolerance, and improved yield and end‐use quality in this population.

## AUTHOR CONTRIBUTIONS

**John H. Price**: Conceptualization; data curation; formal analysis; investigation; methodology; visualization; writing—original draft; writing—review and editing. **Mary J. Guttieri**: Conceptualization; investigation; project administration; resources; supervision; writing—original draft; writing—review and editing. **Moses Nyine**: Methodology; writing—review and editing. **Eduard Akhunov**: Methodology; resources; writing—review and editing.

## CONFLICT OF INTEREST STATEMENT

The authors declare no conflicts of interest.

## Supporting information



Supplemental Figure 1: Visual description of breeding scheme used for population development

Supplemental Table 1: Passport data for all wild emmer parental accessions
Supplemental Table 2: Proportion of RILs with wild emmer introgressions at each chromosome
Supplemental Table 3: Proportion of RILs with wild emmer introgressions at each chromosome arm

## Data Availability

Limited quantities of introgression RILs may be obtained from the Kansas State University WGRC under Material Transfer Agreement. Estimated wild emmer allele proportions at each 1 Mb bin for each RIL line may be obtained from AgDataCommons, Dataset DOI: 10.15482/USDA.ADC/28629710. All sequence data may be accessed from NCBI, BioProject PRJNA1260574.
