# Supplementary material for: Development and characterization of a wild emmer wheat backcross introgression population for hard winter wheat improvement
Source: Plant Genome. 2025 Sep 1;18(3):e70104. doi: 10.1002/tpg2.70104 (PMC12402295; doi:10.1002/tpg2.70104)
Supplement: Supplementary file 1 [file TPG2-18-e70104-s002.pdf]

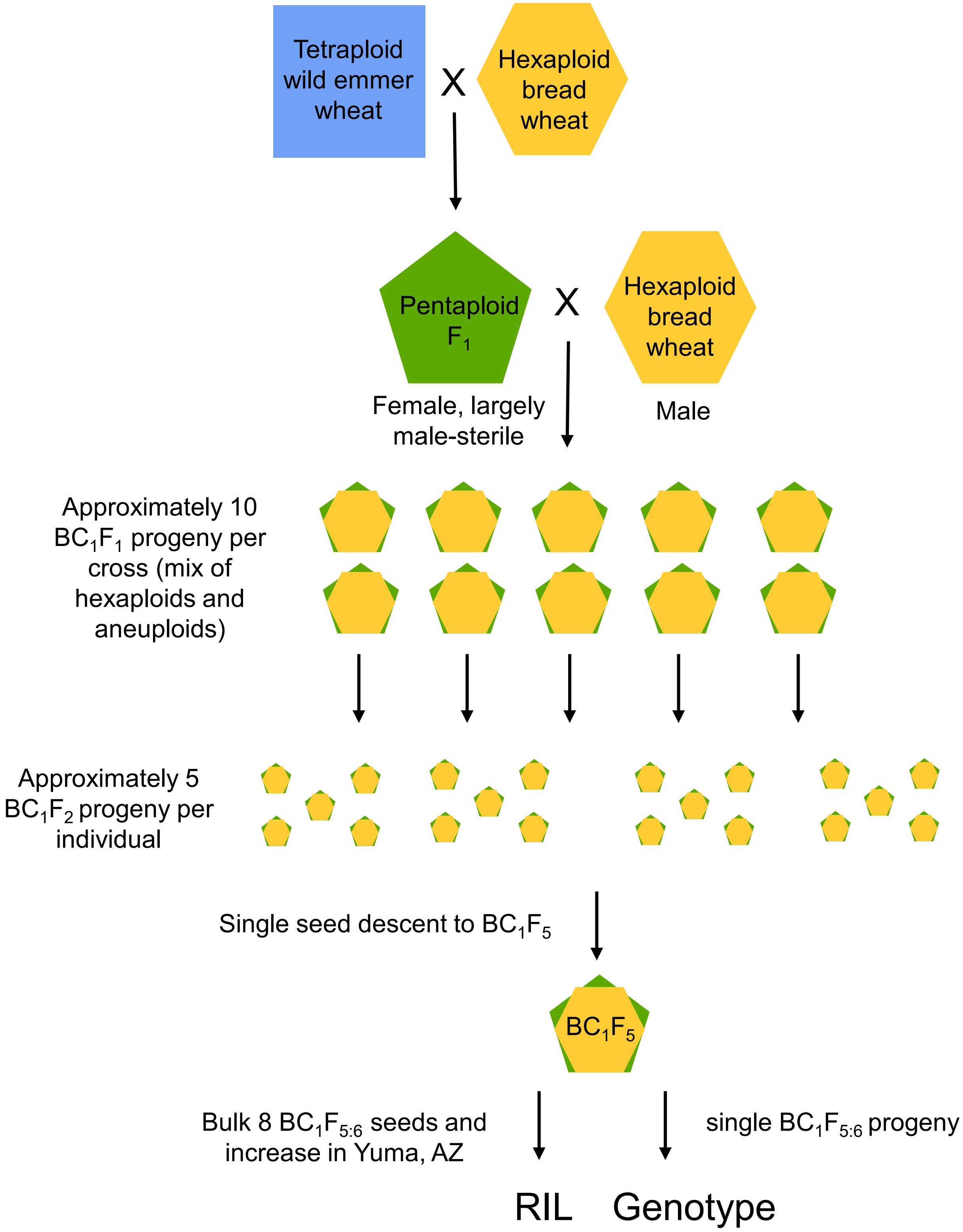

Tetraploid wild emmer wheat
X
Hexaploid bread wheat
Pentaploid F1
X
Hexaploid bread wheat
Female, largely male-sterile
Male
Approximately 10 BC1F1 progeny per cross (mix of hexaploids and aneuploids)
Approximately 5 BC1F2 progeny per individual
Single seed descent to BC1F5
BC1F5
Bulk 8 BC1F5:6 seeds and increase in Yuma, AZ
single BC1F5:6 progeny
RIL
Genotype
