# Supplementary figures and images for: Development and characterization of a wild emmer wheat backcross introgression population for hard winter wheat improvement
Source: Plant Genome. 2025 Sep 1;18(3):e70104. doi: 10.1002/tpg2.70104 (PMC12402295; doi:10.1002/tpg2.70104)

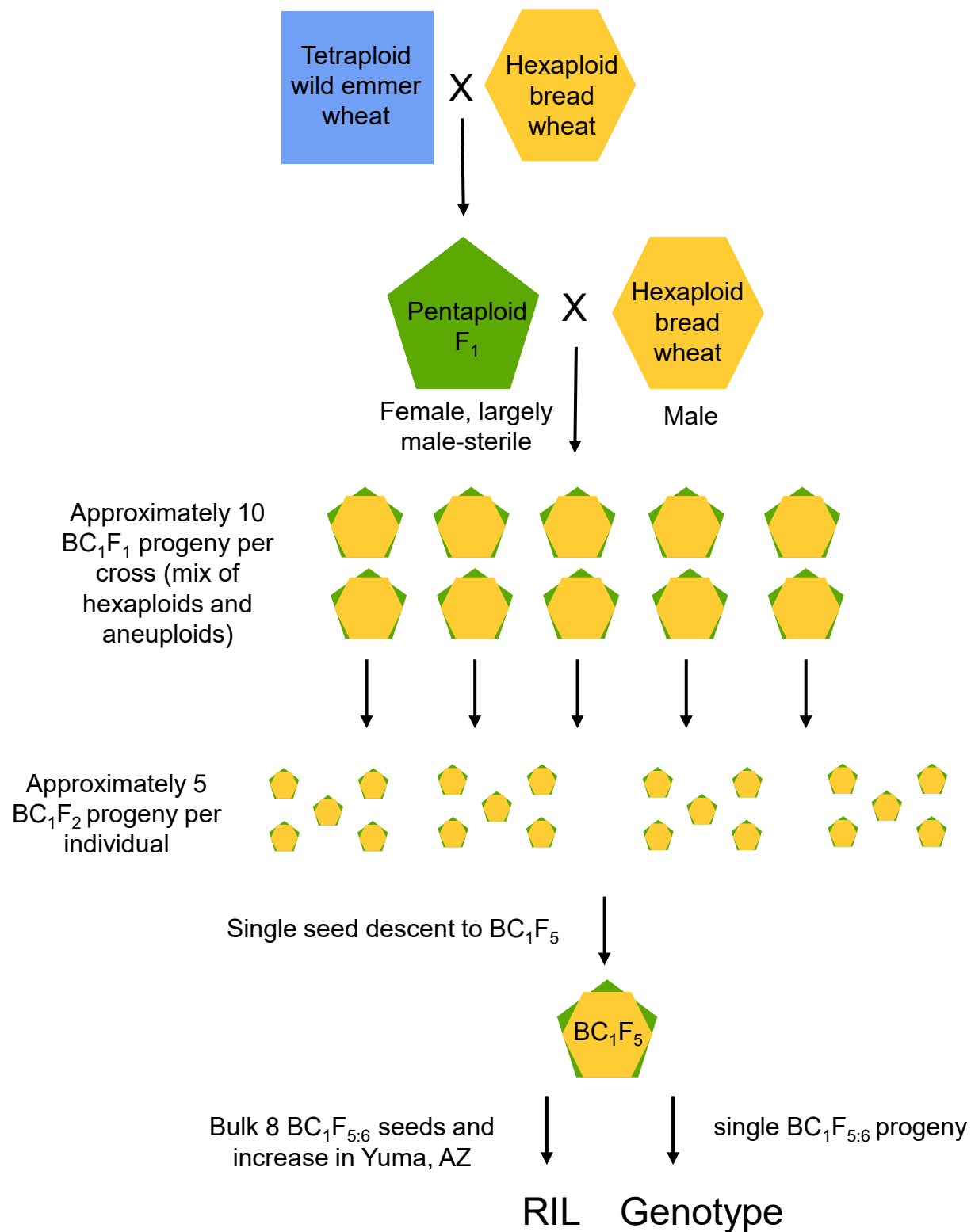

Supplement: Supplementary file 1 — Supplemental Figure 1: Visual description of breeding scheme used for population development [file TPG2-18-e70104-s002.pdf]
